# The Diurnal Timing of Starvation Differently Impacts Murine Hepatic Gene Expression and Lipid Metabolism – A Systems Biology Analysis Using Self-Organizing Maps

**DOI:** 10.3389/fphys.2018.01180

**Published:** 2018-09-10

**Authors:** Christiane Rennert, Sebastian Vlaic, Eugenia Marbach-Breitrück, Carlo Thiel, Susanne Sales, Andrej Shevchenko, Rolf Gebhardt, Madlen Matz-Soja

**Affiliations:** ^1^Rudolf-Schönheimer-Institute of Biochemistry, Faculty of Medicine, Leipzig University, Leipzig, Germany; ^2^Leibniz Institute for Natural Product Research and Infection Biology, Hans-Knöll-Institute, Jena, Germany; ^3^Institute of Biochemistry, Charité – Universitätsmedizin Berlin, Berlin, Germany; ^4^Berlin Institute of Health, Freie Universität Berlin, Humboldt-Universität zu Berlin, Berlin, Germany; ^5^Max Planck Institute of Molecular Cell Biology and Genetics, Dresden, Germany

**Keywords:** hepatocyte, circadian regulation, self-organizing map, starvation, refeeding

## Abstract

Organisms adapt their metabolism and draw on reserves as a consequence of food deprivation. The central role of the liver in starvation response is to coordinate a sufficient energy supply for the entire organism, which has frequently been investigated. However, knowledge of how circadian rhythms impact on and alter this response is scarce. Therefore, we investigated the influence of different timings of starvation on global hepatic gene expression. Mice (*n* = 3 each) were challenged with 24-h food deprivation started in the morning or evening, coupled with refeeding for different lengths and compared with *ad libitum* fed control groups. Alterations in hepatocyte gene expression were quantified using microarrays and confirmed or complemented with qPCR, especially for lowly detectable transcription factors. Analysis was performed using self-organizing maps (SOMs), which bases on clustering genes with similar expression profiles. This provides an intuitive overview of expression trends and allows easier global comparisons between complex conditions. Transcriptome analysis revealed a strong circadian-driven response to fasting based on the diurnal expression of transcription factors (e.g., *Ppara*, *Pparg*). Starvation initiated in the morning produced known metabolic adaptations in the liver; e.g., switching from glucose storage to consumption and gluconeogenesis. However, starvation initiated in the evening produced a different expression signature that was controlled by yet unknown regulatory mechanisms. For example, the expression of genes involved in gluconeogenesis decreased and fatty acid and cholesterol synthesis genes were induced. The differential regulation after morning and evening starvation were also reflected at the lipidome level. The accumulation of hepatocellular storage lipids (triacylglycerides, cholesteryl esters) was significantly higher after the initiation of starvation in the morning compared to the evening. Concerning refeeding, the gene expression pattern after a 12 h refeeding period largely resembled that of the corresponding starvation state but approached the *ad libitum* control state after refeeding for 21 h. Some components of these regulatory circuits are discussed. Collectively, these data illustrate a highly time-dependent starvation response in the liver and suggest that a circadian influence cannot be neglected when starvation is the focus of research or medicine, e.g., in the case of treating victims of sudden starvation events.

## Introduction

Organisms handle periods of food deprivation by drawing on reserves and adapting their metabolism. Humans use fasting to lose weight and for spiritual reasons. The liver is the central hub for metabolic processes, including glucose, amino acid, and lipid metabolism. Therefore, the impact of fasting and refeeding is immense, especially on hepatic parameters (Longo and Mattson, 2014). In this study we addressed the question how the circadian regulation influences the hepatic starvation response with a rarely but powerful used approach called self-organizing map (SOM).

Livers in the post-prandial state store excessive metabolites as glycogen and produce fatty acids, which are transiently stored as triacylglycerides (TAGs) or secreted as very low density lipoprotein (VLDL) (Rui, 2014). The liver synthesizes new carbohydrates and produces ketone bodies in periods of prolonged fasting after depletion of glucose stores. Hormones, such as insulin and glucagon, regulate these processes at a systemic level, and transcription factors, such as PPARs (peroxisome proliferator activated receptor), SREBP transcripts (sterol regulatory element binding transcription protein), and ChREBP [carbohydrate-responsive element-binding protein or MLX-interacting protein-like (MLXIPL)] adjust the metabolism at the molecular level.

Besides the impact of feeding state on the liver metabolism, the timing of food supply influences the metabolic state enormously (Wehrens et al., 2017). In general, the mammalian physiology is synchronized by an inner time-keeping mechanism called the circadian clock. The molecular circadian regulation is based on central clock genes expressed in almost all tissues and cells. The translated proteins generate and regulate the circadian rhythm via transcriptional and translational feedback loops. The transcriptional activators ARNTL (aryl hydrocarbon receptor nuclear translocator-like protein 1 or BMAL1) and CLOCK (circadian locomotor output cycles kaput) stimulate the expression of the negative regulators Period (*Per1*, *Per2*, and *Per3)* and Cryprochrome (*Cry1* and *Cry2*), which in turn repress ARNTL/CLOCK activity. The overall circadian rhythm is coordinated by the suprachiasmatic nuclei (SCN) in the hypothalamus (Ralph et al., 1990; Welsh et al., 2010). Synchronized by an optic light/dark signal the SCN generates output signals coupling the central pacemaker with the peripheral tissues. On the basis of oscillating hormonal and endocrine signals (Yang et al., 2007) the peripheral organs, such as the liver, synchronize their own circadian oscillation. Approximately 15% of all genes are transcribed in a circadian manner whereby one big aspect of circadian regulation includes metabolic processes and energy homeostasis in peripheral tissues, especially in liver (Albrecht, 2012). The tight connection between circadian clock and metabolic processes is underlined by the fact that a vast majority of liver genes is expressed rhythmically, including those regulating, e.g., glucose or lipid metabolism (Stratmann and Schibler, 2006; Ferrell and Chiang, 2015). Interestingly, the liver clock was shown not only be entrained by SCN-synchronization, but also by external factors. One of the most prominent influencers of liver circadian rhythms is the feeding regime. The consumed food volume and the starvation intervals between the meals are able to alter the liver clock (Hirao et al., 2010) and restricted feeding can even rapidly uncouple the liver rhythm from that of SCN (Stokkan et al., 2001).

Disturbance of the normal eating patterns of mice using external food restriction alters the entire metabolism (Jensen et al., 2013), which were analyzed previously using different omics approaches (Bauer et al., 2004; Sokolović et al., 2008; Hakvoort et al., 2011). However, knowledge of specific modulations produced by circadian regulation is scarce. Therefore, we analyzed the impact of fasting and refeeding at two different time-points [zeitgeber time (ZT) 3 and ZT 12] on the physiological and metabolic states of primary hepatocytes at a global omics level. Evaluations of transcriptome data were performed using SOMs. SOMs are an alternative approach that allows the identification of global expression trends via clustering of similarly expressed genes. The relative expression of gene groups is color-coded and enables an intuitive and unbiased interpretation of the data (Wirth et al., 2011). The results demonstrated that the timing of food restriction altered the influence of starvation on hepatocytes. A 24-h starvation period produced different gene expression patterns and lipidome profiles in liver cells depending on the starting time. While starvation started in the morning led to the known adaptions like initiation of gluconeogenesis and suppression of fatty acid and cholesterol synthesis, starvation started in the evening decreased gluconeogenesis-associated gene expression and induced fatty acid synthesis genes. Refeeding mice for 12 h after a 24-h long starvation period was not sufficient to restore the gene expression pattern of *ad libitum* fed mice, which was revealed by persistent dysregulation of essential metabolic pathways, such as lipid metabolism and autophagy. However, an extended refeeding period of 21 h approached an expression pattern similar to that of the *ad libitum* state, but some differences persisted.

## Materials and Methods

### Maintenance of the Mice and Feeding

Male C57BL/6N mice were maintained in a pathogen-free facility on a 12:12 h light–dark cycle (light on at 6 a.m = ZT 0, light off at 6 p.m. = ZT 12), according to German guidelines and those of the world medical association declaration of Helsinki for the care and safe use of experimental animals. The animal experiments were approved by the Landesdirektion Sachsen. The mice had free access to regular chow (ssniff^®^ R/M-H V1534; 58%, 33%, 9% calories from carbohydrates, proteins and fat, respectively; metabolisable energy: 12.8 kJ/g; ssniff^®^ Spezialdiaeten GmbH, Germany) and tap water.

**FIGURE 1:**
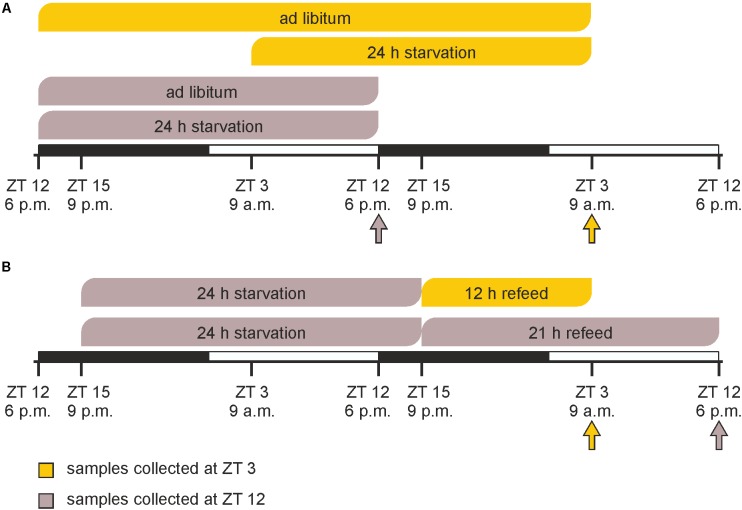
Timeline of feeding/starving cycles and sacrifice. **(A)** Mice were fed *ad libitum* or starved for 24 h prior to sacrifice at ZT 3 (9 a.m.) or ZT 12 (6 p.m.). **(B)** Mice were starved for 24 h from ZT 15 (9 p.m.) to ZT 15 and refed for 12 or 21 h prior sacrifice. The arrows indicate the times of sacrifice.

Prior to the experimental procedures, animals were randomly segregated into six groups (*n* = 3 each). In a first experiment, starvation was started either at ZT 3 (9 a.m.) or at ZT 12 (6 p.m.) and mice were sacrificed after 24 h at the same times on the next day together with *ad libitum* fed groups (**Figure 1A**). In the refeeding experiment two groups of mice were starved for 24 h started at ZT 15 followed by refeeding for 12 or 21 h until ZT 3 and ZT 12 on the next day, respectively (**Figure 1B**).

### Isolation of Primary Mouse Hepatocytes, RNA Isolation, and Quantitative Real-Time PCR (qPCR)

The primary hepatocytes were isolated from male C57BL/6N mice, treated like explained above. Isolation was performed by a collagenase perfusion technique as described before (Gebhardt et al., 2003). The cell suspension was cleared of non-parenchymal cells by differential centrifugation steps (Matz-Soja et al., 2014). Pure hepatocyte fraction was used for further procedures.

Total RNA from hepatocytes was extracted using RNeasy^®^ Mini Kit (Qiagen, Hilden) and the quality was controlled by agarose gel electrophoresis. The reverse transcription was performed with the Proto Script M-MuLV First Strand cDNA Synthesis Kit (New England Biolabs). Gene expression quantification by qPCR was performed in duplicates using the Rotor-Gene SYBR^®^ Green PCR Kit and a Rotor-Gene Q (Qiagen). Gene specific intron-spanning primers were designed with Primer 3 software and are listed in **Supplementary Table 1**. The specific qPCR products were quantified using internal amplification standards; 18S was used as reference gene. Values are plotted as average of biological replicates (*n* = 3) ± standard deviation. The statistical evaluation was performed with the unpaired Student’s *t*-test (GraphPad Prism 7). The null hypothesis was rejected at ^∗^*p* < 0.05, ^∗∗^*p* < 0.01, and ^∗∗∗^*p* < 0.001 levels.

### Illumina Microarray Processing and Data Analysis Using SOM

For each condition, the RNA from three mice was pooled and used as one sample in the microarrays (BeadChip Array MouseRef-8 v2, Illumina). The analysis was performed by the Interdisciplinary Centre for Clinical Research, Leipzig (Faculty of Medicine, Leipzig University). The following link provides the raw data of the microarray^1^.

All computational analyses were performed using R software (Ihaka and Gentleman, 1996). Microarrays were annotated using the annotation provided by Illumina and raw expression data were pre-processed using the lumi package (Du et al., 2008). Pre-processing included background correction using the bgAdjust method, variance stabilization transformation, and quantile normalization. Detection calls were performed with default parameters to remove absent beads. Furthermore, the expression values for single beads were mapped to a gene symbol identifier. For each array, multiple bead IDs mapping to the same gene symbol were averaged.

General expression trends in each sample were identified using SOMs as implemented in the oposSOM package (Löffler-Wirth et al., 2015). SOMs are artificial neural networks trained by unsupervised learning (Kohonen, 1982). This machine learning approach allows dimension reduction of high-dimensional data by clustering similarly regulated genes to so-called “metagenes”. For each treatment, it can be defined if the expression of a certain metagene is over or under the mean expression of this metagene in the analyzed pool (termed as over- and underexpression), and all groups are compared equivalently. The metagenes, here 20 × 20, can be visualized in a map where adjacent metagenes have similar expression profiles and more distant ones are expressed differently, with the invariant genes located in the center of the map. Furthermore, a color gradient codes the expression level of the metagenes, maroon indicating the highest expression, blue the lowest expression, and yellow and green intermediate levels (Wirth et al., 2011). This image-based analysis allows an intuitive interpretation and provides an overview of the overall regulation patterns. Apart from the creation of SOMs, the oposSOM package also provided additional analytical methods including the identification of over- or underexpressed spots in the SOMs, clustering of metagenes using k-means (Hartigan and Wong, 1979), and enrichment testing for metagene-cluster and over- or underexpressed spots using Gene Ontology (GO) terms (Ashburner et al., 2000) as provided by the Ensembl database (Aken et al., 2016).

Based on the k-means clustering as performed automatically by the oposSOM package, we performed a gene enrichment analysis (GEA) for all genes with an absolute expression change ≥ 1.5-fold and the genes from each cluster using the web-based tool DAVID (Database for Annotation, Visualization and Integrated Discovery Bioinformatics Resources 6.8^2^, **RRID**:SCR_001881). We selected *Mus musculus* as the background and filtered the GO terms with a Benjamini–Hochberg corrected *p*-value < 0.05.

Visualization of single genes for the identified significant GO terms was performed using heatmaps. The expression levels of single genes were displayed as relative expression values compared to the mean expression in all six samples analogous to the metagenes (termed as over- and underexpression). All genes with an absolute expression change ≥ 1.5-fold [log_2_(1.5)] between at least two of the six groups were considered for further analysis.

The STRING database version 10.5^3^ was used to explore the interactions between the studied genes/proteins related to hepatic starvation response (Szklarczyk et al., 2015). The networks were constructed in the “confidence” mode with a high confidence score (0.7).

### Shotgun Lipidomics

Lipids from primary hepatocytes were extracted by a modified protocol of Folch (Folch et al., 1957) and analyzed by shotgun mass spectrometry as described previously (Schuhmann et al., 2012). Briefly, hepatocytes (an amount equivalent to 10 μg of total protein) were dissolved in 200 μl ammonium bicarbonate solution (150 mM). For the subsequent quantification 10 μl internal standard mixture were added (20 pmol TAG 12:0-12:0-12:0, 20 pmol DAG 17:0- 17:0, 40 pmol diethyl PC 18:0-18:0, 50 pmol diethyl PE 20:0-20-0, 10 pmol PG 17:0-17-0, 40 pmol PS 12:0-12:0, 50 pmol PI 16:0-16-0, 40 pmol LPC 12:0, 40 pmol LPE 14:0, 30 pmol SM d18:1-12:0, 90 pmol CE 12:0, 20 pmol Cer d18:1-12:0, 50 pmol cholesterol d7; Avanti Polar Lipids, Inc., Alabaster, AL, United States). Then, 265 μl of methanol and 730 μl of chloroform were added and the mixture was vortexed for 1 h at 4°C. The lower organic phase was collected, dried in a vacuum centrifuge and the lipid extracts were re-dissolved in 120 μl chloroform:methanol [1:2 (v/v)] mixture. The analysis was performed in both, negative, and positive ion mode. For negative mode analyses, 10 μl extract were mixed with either 12 μl of 13 mM ammonium acetate in isopropanol or 0.1% (v/v) triethylamine in methanol. For positive mode analyses, 10 μl extract were mixed with 90 μl of 6.5 mM ammonium acetate in isopropanol before infusion. The analyses were performed on a Q Exactive mass spectrometer (Thermo Fisher Scientific, Germany) equipped with a robotic nanoflow ion source TriVersa NanoMate (Advion BioSciences, Ithaca, NY, United States). High resolution (140,000 at m/z 200) FT-MS spectra were acquired for 1 min within the range of m/z 420–1000 in negative and 450–1000 in positive mode. Cholesterol was quantified as previously described (Liebisch et al., 2006). Briefly, 30 μl of extract were dried under vacuum, then 75 μl acetyl chloride:chloroform [1:2 (v/v)] were added, incubated for 1 h at room temperature, dried under vacuum and re-dissolved in 60 μl chloroform:methanol [1:2 (v/v)]. 10 μl extract were mixed with 90 μl of 6.5 mM ammonium acetate in propanol before infusion and analyzed in positive ion mode. The following lipid classes were identified and quantified using the LipidXplorer software (Herzog et al., 2011): tri- and diacylglycerides (TAG/DAG), cholesteryl esters (CE), sphingomyelins (SM), phosphatidylethanolamines (PEs), phosphatidylcholines (PCs), and cholesterol. The concentrations are plotted in pmol lipid per μg total protein as average of the biological replicates (*n* = 3) ± standard deviation.

## Results

Feeding procedures immensely affect an organism’s metabolism. The present study examined alterations in the physiology and the metabolism of murine hepatocytes following a 24-h starvation period initiated at ZT 3 (morning) or ZT 12 (evening) compared to hepatocytes from mice fed *ad libitum* (**Figure 1A**). Our data indicated that the effects strongly depended on the timing of starvation. We also compared hepatocytes in refed state to the corresponding starvation group and *ad libitum* fed mice to evaluate whether 12 or 21 h of refeeding were sufficient to restore the *ad libitum* expression profile (**Figure 1B**).

### Global Hepatic Gene Expression Is Influenced by Feeding and Timing

**FIGURE 2:**
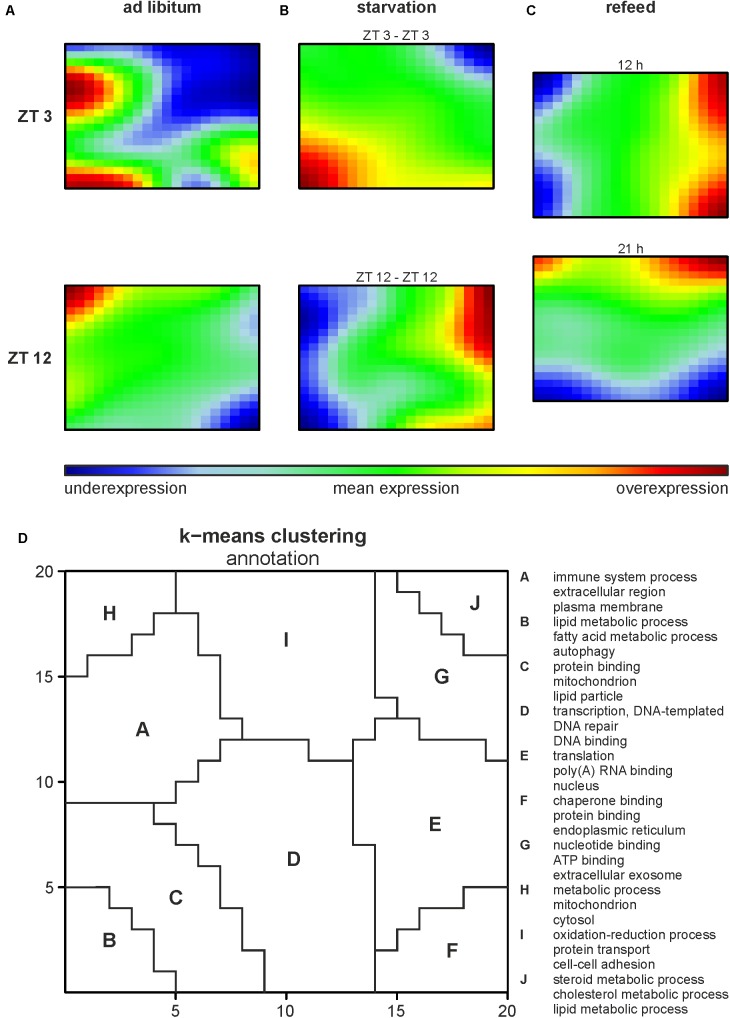
Global hepatic gene expression dependent on feeding patterns and timing. **(A–C)** Self organizing maps (SOMs) illustrating the expression of metagenes (20 × 20); maroon indicates an overexpression, blue an underexpression. Mice were sacrificed at ZT 3 and ZT 12 after **(A)** fed *ad libitum*, **(B)** starved for 24 h or **(C)** starved for 24 h and refed for 12 h until ZT 3 or 21 h until ZT 12. **(D)** Annotation of the SOMs using k-means clustering and following gene-enrichment analysis. Selected GO terms are shown. A full list is available in **Supplementary Table 2B**.

Illumina microarrays, analyzed, and visualized using SOMs, delivered an overview of the hepatic alterations induced by different feeding regimes. A GEA of all regulated genes (**Supplementary Table 2A**) produced an impression of the involved GO terms. Further we performed a separate analysis for each cluster to distinguish between them. Based on the SOMs of the *ad libitum* samples from ZT 3 and ZT 12 it strikes that a large amount of the genes in the liver were expressed in a circadian manner (**Figure 2A**), since the expression of almost all metagenes differed. ZT 3 showed two overexpression spots at the left margin, annotated as clusters A and B by a k-means clustering of the SOMs (**Figure 2D** and **Supplementary Table 2B**). Cluster A is associated with immune system processes (‘complement activation,’ classical pathway,’ ‘innate immune response’) and many GO terms in regard to extracellular space and membrane (‘extracellular region,’ ‘plasma membrane,’ ‘cell surface’), while cluster B contains many lipid metabolism associated GO terms (‘fatty acid metabolic process,’ ‘acyl-CoA metabolic process’) and the GO term ‘autophagy.’ The large underexpression spot in the upper right corner partly covered by cluster J contains many cholesterol and steroid associated GO terms (‘cholesterol metabolic process,’ ‘steroid metabolic process,’ ‘fatty acid metabolic process’). In comparison to ZT 3, ZT 12 exhibited a mean expression in the area of clusters B and J (**Figures 2A,D**). But ZT 12 had a small overexpressed area in the upper left corner (cluster H) related to the GO term ‘metabolic process’ and an underexpression spot in the lower right corner (cluster F) containing GO terms associated with protein binding and degradation (‘chaperone binding,’ ‘endoplasmic reticulum,’ ‘proteasome accessory complex’).

Starvation started at both ZT 3 and ZT 12 immensely changed the expression profiles of the metagenes in the SOMs compared to the related *ad libitum* state (**Figure 2B**). Additionally, comparing ZT 3 and ZT 12 starvation no congruence is visible, which implies a diurnal-driven response to fasting. The 24-h starvation period at ZT 3 began during the day followed by a whole night of fasting (**Figure 1A**), which changed the size and shape of the overexpression spot in the lower left corner and the underexpression spot in the upper right corner (clusters B and J), regions associated with lipid and steroid metabolism (**Figures 2B,D**). In contrast, the ZT 12 starvation period started in the night followed by a whole day of fasting (**Figure 1A**) and resulted in a totally changed metagene expression, where the whole left margin is underexpressed, while the right margin shows an overexpression (**Figure 2B**). The regions (clusters B and J) where GO terms related to autophagy, lipid and steroid metabolism are localized have an opposed expression comparing ZT 3 and ZT 12 starvation.

The refeeding experiment (**Figure 1B**) revealed disparate SOMs after refeeding mice for 12 h and the ZT 3 *ad libitum* group (**Figure 2C**). However, the SOM of 12 h refeed (**Figure 2C**, upper panel) showed high similarity with the corresponding ZT 12 starvation group (**Figure 2B**, lower panel). The mice sampled at ZT 12 were starved for the same 24-h period, but were refed for 21 h prior sacrifice. This prolonged refeed period produced a somehow mixture of expression profiles from ZT 12 *ad libitum* and starvation state (**Figure 2C**). While clusters F and H resemble that of ZT 12 *ad libitum* (**Figure 2A**, lower panel), the regions where lipid and steroid metabolism are localized (clusters B and J) still showed the starvation pattern (**Figure 2B**, lower panel).

### Alterations in Relevant Metabolic Features Following Starvation and Refeeding

SOM analysis identified differently regulated metabolic alterations produced by the various feeding regimes. A list of all regulated genes detected by the microarrays is compiled in **Supplementary Table 3**. The present study focused on the expression of genes related to intermediary metabolism (glucose and lipid metabolism), steroid metabolism, insulin signaling, and autophagy. We also analyzed central clock gene expression because the circadian aspect of the feeding regimes was particularly interesting. The interactions of the involved genes/proteins were visualized within a protein interaction network, illustrating the mutual connections (**Supplementary Figure 1**).

#### Circadian Regulation

##### Diurnal influence

**FIGURE 3:**
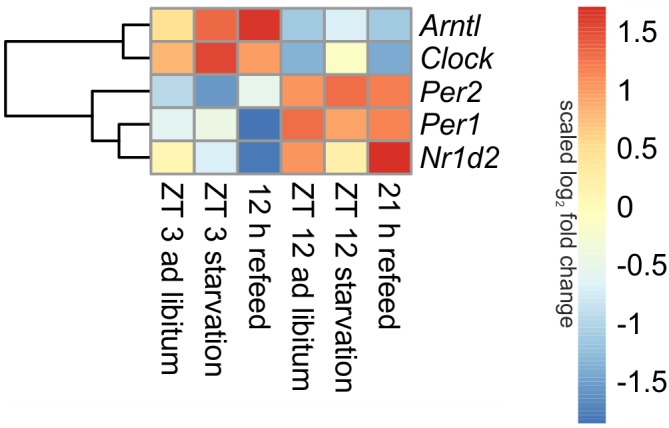
Heatmap of differentially expressed central circadian genes. Red indicates an overexpression, and blue indicates an underexpression.

Being the first order clock genes, *Arntl* and *Clock* gene products activate the expression of different target genes, including *Per1*/*2*/*3* homolog and *Cry*. PER and CRY, in turn, repress their own expression by interacting with ARNTL and CLOCK (Partch et al., 2014). Consequently to these regulations the expression of *Arntl*/*Clock* and *Per* occurs in antiphase, which is seen in **Figure 3** (*ad libitum*). In the *ad libitum* state *Arntl* and *Clock* were overexpressed at ZT 3 and underexpressed at ZT 12, *Per1* and *Per2* were regulated vice versa. *Nr1d2* (Rev-erb beta), another gene of the negative feedback loop, exhibited a typical rhythmic regulation with a higher expression at ZT 12 than at ZT 3. These results were verified by qPCR and used to validate the microarray (data not shown).

##### Starvation

Expression of central clock genes were primarily regulated in a diurnal manner (**Figure 3**). However, fasting also influenced central circadian genes. A 24-h starvation period initiated at ZT 3 or ZT 12 increased *Arntl* and *Clock* expression levels, but the typical diurnal regulation of these genes remained. The opponent *Per2* was down-regulated after starvation initiated at ZT 3 and up-regulated in the ZT 12 starvation mice compared to the corresponding *ad libitum* samples. The *Per1* expression was only marginally changed by 24-h starvation in the morning and the evening, while *Nr1d2* was decreased after both starvation periods.

##### Refeed

When mice were fasted for 24 h and refed for 12 h until ZT 3, the *Arntl* expression was highly increased, while a prolonged refeed period of 21 h abolished the effect of starvation (**Figure 3**). The *Clock* expression was no longer altered after both refeed periods. *Per1* expression was decreased after a 12 h refeed, but the 21 h refeed already restored the *ad libitum* expression. *Per2* expression was higher after both refeed periods in comparison to *ad libitum*. The expression of *Nr1d2* showed big changes after both refeed periods, the expression was further decreased after 12 h and increased after 21 h refeed.

All in all, a 21 h refeeding seemed to be sufficient to abolish the alterations in the expression of the genes of the primary negative-feedback loop (*Arntl*, *Clock*, *Per1*, and *Per2*) caused by starving the mice. But after 12 h refeed the expression of *Arntl*, *Per1*, and *Per2* still showed major changes compared to the *ad libitum* sample.

#### Insulin Signaling

##### Diurnal influence

**FIGURE 4:**
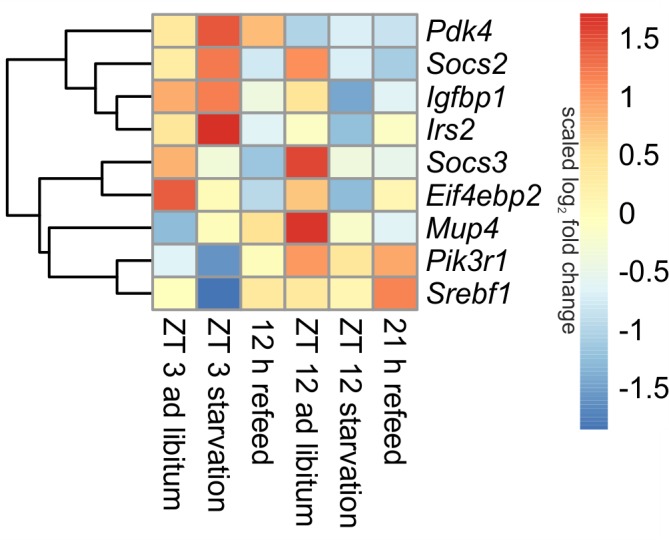
Heatmap of differentially expressed genes related to insulin signaling. Red indicates an overexpression, and blue indicates an underexpression.

Insulin is one of the master regulators of the metabolism and its effects are partially mediated by insulin receptor signaling (Boucher et al., 2014). The microarray analyses revealed diurnal regulation of several genes of this pathway (**Figure 4**). For example, *Pik3r1* (phosphatidylinositol 3-kinase, regulatory subunit, polypeptide 1), part of the inositol phosphorylation complex, showed a lower expression in the morning (ZT 3) compared to the evening (ZT 12). The same regulation occurs for *Mup4* (major urinary protein 4) whose influence on energy metabolism is not well understood yet. However, *Pdk4* (pyruvate dehydrogenase kinase, isoenzyme 4), regulator of glucose metabolism, was higher expressed in the morning than in the evening.

##### Starvation

The 24-h starvation period initiated in the morning produced strong overexpression of central hepatic insulin signaling genes [e.g., *Pdk4*, *Mup4*, *Irs2* (insulin receptor substrate 2) and *Igfbp1* (insulin like growth factor binding protein 1)]. Notably, all of these genes, except *Pdk4*, were strongly down-regulated after starvation initiated at ZT 12. The expression of *Pik3r1* and *Eif4ebp2* (eukaryotic translation initiation factor 4E binding protein 2), which is a regulator of translation, was lowered in both starvation periods (**Figure 4**). The transcription factor *Srebf1c* was down-regulated only due to starvation started at ZT 3, and it was unaffected by evening starvation (**Figure 6**).

*Socs2* and *Socs3* (suppressor of cytokine signaling), which were not regulated diurnally in *ad libitum* fed mice, are associated to the negative regulation of insulin signaling. The data for *Socs2* and *Socs3* revealed a change from an overexpression in ZT 12 *ad libitum* sample to a strong underexpression due to starvation started at ZT 12. *Socs3* expression also slightly decreased when starvation was initiated at ZT 3, but *Socs2* expression slightly increased (**Figure 4**).

##### Refeed

The expression of nearly all detected genes of insulin receptor signaling did not recover during the 12 h refeeding and resembled the expression of the ZT 12 starvation mice (**Figure 4**). Exceptions included *Pdk4*, which almost reached *ad libitum* levels, and *Srebf1c*, which was highly induced during refeeding. The gene expression levels of *Pdk4*, *Eif4ebp2*, *Pik3r1*, and *Irs2* after a refeeding period of 21 h were similar to the corresponding *ad libitum* levels. The *Socs2* and *Socs3* genes were considerably underexpressed after both refeeding periods.

#### Autophagy

##### Diurnal influence

**FIGURE 5:**
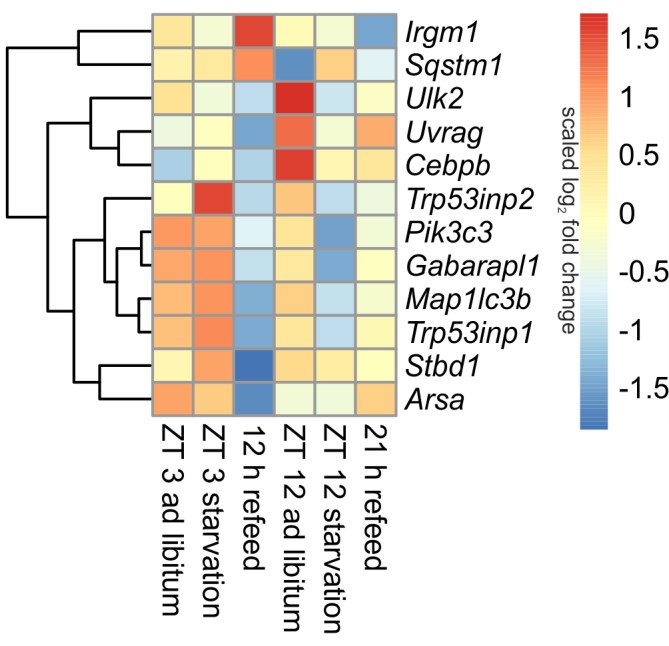
Heatmap of differentially expressed genes related to autophagy. Red indicates an overexpression, and blue indicates an underexpression.

In *ad libitum* fed mice, central genes of the mammalian autophagy pathway, such as *Ulk2* (unc-51 like kinase 2) and *Uvrag* (UV radiation resistance associated gene), were lower expressed in the morning compared to evening. However, *Sqstm1* (sequestosome 1), which is required for the recognition of protein aggregates by the autophagy machinery (Bjorkoy et al., 2005), was significantly down-regulated at ZT 12 compared to ZT 3 *ad libitum*. A regulator of autophagy, *Cebpb* (CCAAT/enhancer binding protein beta) was higher expressed in the evening than in the morning (**Figure 5**).

##### Starvation

When starvation was started at ZT 3 expression of many of the autophagy-associated genes [e.g., *Ulk2*, *Uvrag*, *Sqstm1*, *Pik3c3* (phosphatidylinositol 3-kinase catalytic subunit type 3), *Trp53inp1* (transformation related protein 53 inducible nuclear protein 1), *Gabarapl1* (gamma-aminobutyric acid A receptor-associated protein-like 1), *Map1lc3b* (microtubule-associated protein 1 light chain 3 beta)] was not altered (**Figure 5**). Only *Trp53inp2*, which is essential for autophagosome formation, was markedly up-regulated during the ZT 3 starvation period.

This observation dramatically changed when the starvation period of 24-h was started at ZT 12. The above-mentioned central autophagy genes, with the exception of *Sqstm1*, were dramatically down-regulated during this fasting period, which was especially prominent for *Ulk2* expression. The transcription factor *Cebpb* was up-regulated after food deprivation started at ZT 3 and down-regulated in the evening (**Figure 5**).

##### Refeed

Our study revealed that the dramatic down-regulation of central autophagy genes due to the initiation of starvation in the evening remained during a refeeding period of 12 h (**Figure 5**). The expression of *Uvrag*, *Trp53inp1*, *Stbd1* (starch binding domain 1) and *Arsa* (arylsulfatase A) was further reduced. However, *Sqstm1* and *Irgm1* (immunity-related GTPase family M member 1), which are involved in autophagic protein degradation (Traver et al., 2011), were up-regulated during the 12 h refeeding period. After a prolonged refeeding of 21 h, most autophagy-related genes approached the ZT 12 *ad libitum* expression levels, but did not reach it. *Cebpb* was expressed at the *ad libitum* level after the 12 h refeed, but its expression was lowered after the 21 h refeed (**Figure 5**).

#### Intermediary Metabolism

##### Diurnal influence

**FIGURE 6:**
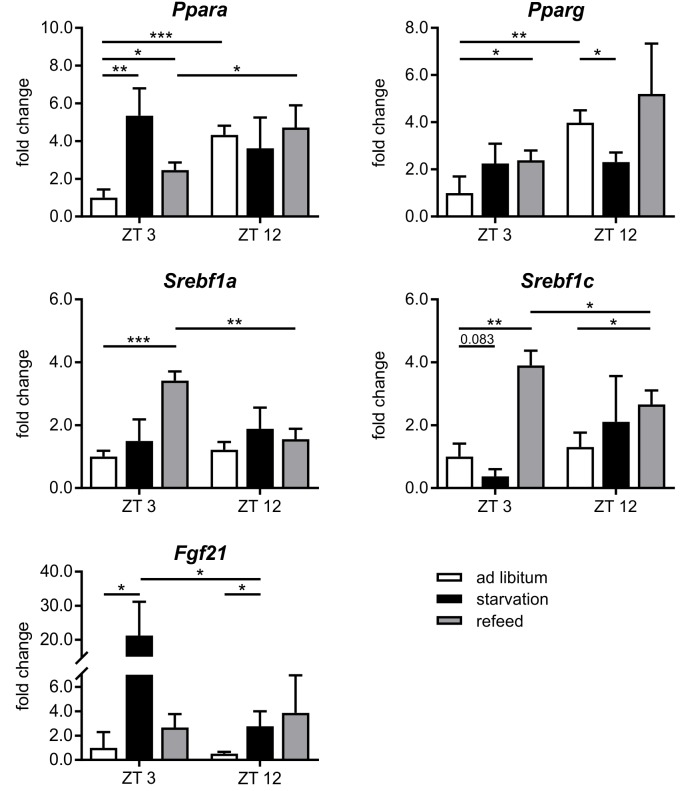
Response of regulatory factors to starvation and refeeding in a diurnal manner. Expression of *Ppara*, *Pparg*, *Srebf1a*, *Srebf1c*, and *Fgf21* in primary hepatocytes of mice fed *ad libitum* (white bars), starved 24 h (black bars) or starved 24 h and refed 12 h (until ZT 3) and 21 h (until ZT 12) (gray bars) quantified using qPCR (*n* = 3). Data are plotted as mean ± standard deviation relative to ZT 3 *ad libitum*, ^∗^*p* < 0.05, ^∗∗^*p* < 0.01, and ^∗∗∗^*p* < 0.001.

**FIGURE 7:**
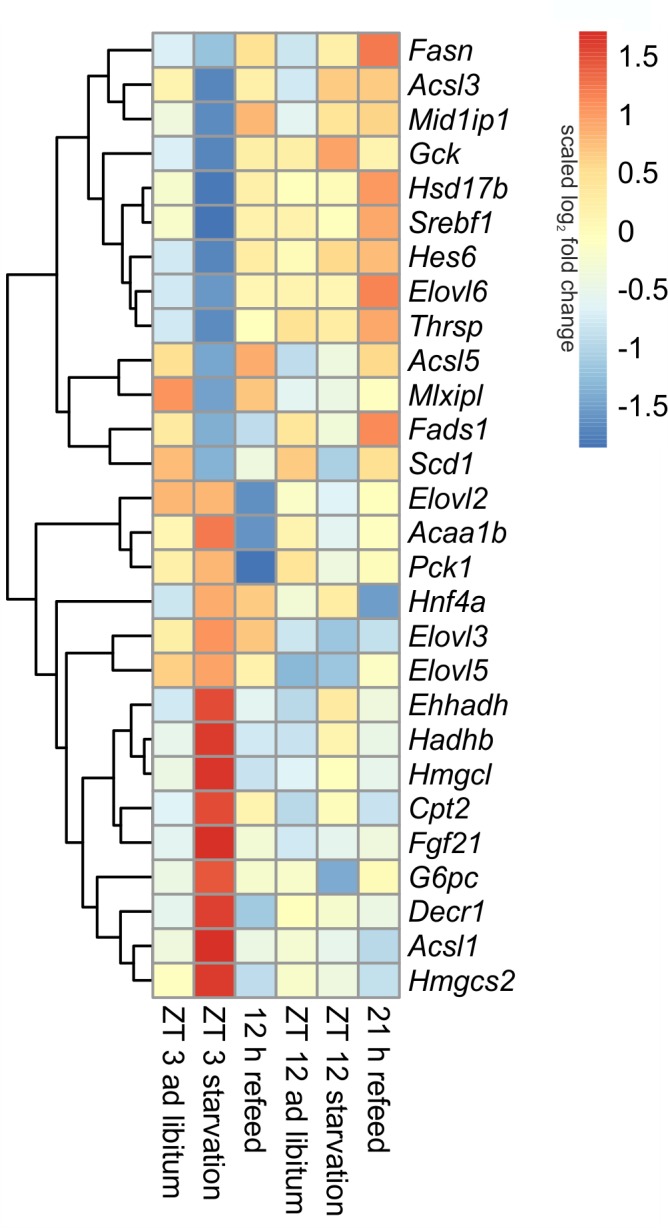
Heatmap of differentially expressed genes related to intermediary metabolism. Red indicates an overexpression, and blue indicates an underexpression.

Many genes associated with intermediary metabolism (lipid and glucose metabolism) showed a time-dependent expression. The transcription factors that mainly regulate intermediary metabolism, such as *Ppara*, *Pparg*, and *Hnf4a* (hepatocyte nuclear factor 4 alpha), were higher expressed at ZT 12 than at ZT 3, *Mlxipl* (ChREBP) exhibits a vice versa regulation (**Figures 6, 7**). *Srebf1* transcripts showed no diurnal changes (**Figure 6**). The members of the Elovl (elongation of very long chain fatty acids protein) family (*Elovl2*, *Elovl3*, and *Elovl5*), which are involved in lipid metabolism, were higher expressed at ZT 3 compared to ZT 12, but *Elovl6* exhibited an inverse regulation (**Figure 7**).

##### Starvation

The regulation of hepatic lipid and glucose metabolisms is based on different transcription factors. The expression of *Ppara, Pparg*, and *Hnf4a* was elevated after starvation initiation at ZT 3 compared to the *ad libitum* group (**Figures 6, 7**).

Notably, starvation initiated at ZT 12 decreased the expression of *Pparg* by significantly by more than 40% and *Ppara* and *Hnf4a* were only marginally regulated. *Hes6*, which is an interaction partner of HNF4a, exhibited diminished expression during starvation started at ZT 3 and increased expression under ZT 12 starvation. *Fgf21* (fibroblast growth factor 21), which is another regulatory element that is closely connected to the PPAR family and the starvation response, exhibited an expression increase by approximately 20-fold after starvation initiated at ZT 3, but this increase was much lower in the ZT 12 group (**Figure 6**). *Mlxipl* was strongly down-regulated after starvation in the morning and was not altered in the evening group. The *Srebf1c* mRNA levels decreased by more than threefold after food deprivation started ZT 3 but were not affected in the ZT 12 group.

The liver ensures the synthesis of sufficient amounts of acetyl coenzyme A (CoA), especially during starvation. Therefore, beta-oxidation is increased due to starvation by the up-regulated expression of many of the involved enzymes, including *Cpt2* (carnitine palmitoyltransferase 2), *Hadhb* (hydroxyacyl-CoA dehydrogenase/3-ketoacyl-CoA thiolase/enoyl-CoA hydratase), *Ehhadh*, *Acaa1b* (acetyl-CoA acyltransferase 1B), *Acsl1* (acyl-CoA synthetase long-chain family member 1) and *Decr1* (2,4-dienoyl CoA reductase 1) (**Figure 7**). However, this increase was much greater when starvation was initiated at ZT 3 rather than at ZT 12. Members of the *Acsl* family are responsible for the activation of long fatty acids and exhibited different regulation. While *Acsl1* strongly increased by starvation started at ZT 3, *Acsl3* and *Acsl5* strongly decreased. In contrast, starting starvation of mice at ZT 12, *Acsl1* and *Acsl5* expression was not altered and *Acsl3* increased.

Ketogenesis is an essential metabolic pathway to generate ketone bodies during starvation. Genes for the two essential enzymes of ketone body formation, *Hmgcs2* (3-hydroxy-3-methylglutaryl-CoA synthase 2) and *Hmgcl* (3-hydroxymethyl-3-methylglutaryl-CoA lyase), were highly increased after 24-h starvation started at ZT 3, but regulation was only marginal at ZT 12. *Hmgcs2*, *Hmgcl*, *Ehhadh*, *Hadhb*, and *Acaa1b* are also involved in the degradation of the amino acids valine, isoleucine, and leucine. The up-regulation of all enzymes after starvation initiated at ZT 3 ensured an efficient supply of acetyl-CoA (**Figure 7**).

The key enzyme of fatty acid synthesis, *Fasn* (fatty acid synthase), was decreased by 24-h starvation started at ZT 3 and, surprisingly, increased in the ZT 12 group. The gene expression of enzymes involved in elongation and formation of unsaturated fatty acids [e.g., *Scd1* (stearoyl-CoA desaturase 1), *Fads1* (fatty acid desaturase 1), *Hsd17b12* (hydroxysteroid-17-beta dehydrogenase 12), *Thrsp* (thyroid hormone responsive), and *Mid1ip1* (Mid1 interacting protein 1)] were predominantly down-regulated after starvation initiated at ZT 3 (**Figure 7**). In contrast, starvation initiated at ZT 12 elevated *Scd1* and *Mid1ip1* expression and did not change *Fads1*, *Hsd17b12*, and *Thrsp*. The members of the Elovl family showed only a regulation when starvation was started at ZT 3, but were not influenced at ZT 12. The *Elovl6* level strongly decreased at ZT 3 starvation, whereas *Elovl3* increased (**Figure 7**).

By gluconeogenesis the liver can synthesize glucose, which is an essential pathway during starvation. As expected, *Pck1* (phosphoenolpyruvate carboxykinase 1), the enzyme for the rate-limiting step of gluconeogenesis, and *G6pc* (glucose-6-phosphatase catalytic subunit), essential to release glucose in the last step of gluconeogenesis, were both increased due to starvation started at ZT 3, but decreased at ZT 12. *Gck* (glucokinase), catalyzing the opposite reaction, shows an inverse regulation (**Figure 7**).

Taken together, the expression patterns at ZT 3 predominantly corresponded to the known starvation responses of the liver. However, the initiation of fasting at ZT 12 revealed unknown regulatory events that produced more subtle and inversely regulated expression levels.

##### Refeeding

Refeeding mice after a 24 h starvation challenges the liver. The depleted stores must be refilled, and the metabolism should return to a normal feeding state. The transcription factors *Ppara* and *Pparg* remained elevated after the 12 h refeeding compared to the *ad libitum* group, but *ad libitum* expression was nearly restored after refeeding for 21 h. *Fgf21* expression remained increased after both refeeding periods (**Figure 6**). By contrast, *Hnf4a* expression after the 12 h refeeding was comparable to that after starvation started at ZT 12 and decreased after the 21 h refeeding compared to *ad libitum*. *Mlxipl* expression was restored after the 12 h refeeding (**Figure 7**). The expression of *Srebf1c* increased significantly after both refeeding periods (**Figure 6**).

Hepatocytes exhibited a strong up-regulation of *Fasn* expression, especially after the 21 h refeed; while after 12 h refeed the increase was comparable with starvation started at ZT 12. The genes *Scd1*, *Acsl5*, *Elovl6*, *Hsd17b12*, *Fads1*, and *Thrsp* showed a similar regulation pattern as *Fasn*. However, the expression of other enzymes, such *Acsl1*, *Acsl3*, *Elovl2*, and *Elovl3* resembled the corresponding *ad libitum* sample after 12 and 21 h refeed (**Figure 7**). Expression of the enzymes of beta-oxidation and mitochondrial fatty acid synthesis were restored (*Cpt2*, *Ehhadh*, *Hadhb*, *Decr1*) or remained decreased (*Acaa1b*) after both refeeding periods (**Figure 7**).

The synthesis of ketone bodies and degradation of amino acids are no longer a necessary energy source when sufficient amounts of nutrients are available. Accordingly, the expression of *Hmgcs2* was slightly decreased after 12 and 21 h of refeeding compared to the corresponding *ad libitum* samples, and *Hmgcl* exhibited the same expression as the *ad libitum* state. The level of *Pck1* strongly decreased after the 12 h refeeding compared to *ad libitum* and was slightly reduced after refeeding for 21 h. Both refeeding times restored *G6pc* expression to *ad libitum* levels (**Figure 7**).

#### Steroid Metabolism

##### Diurnal influence

**FIGURE 8:**
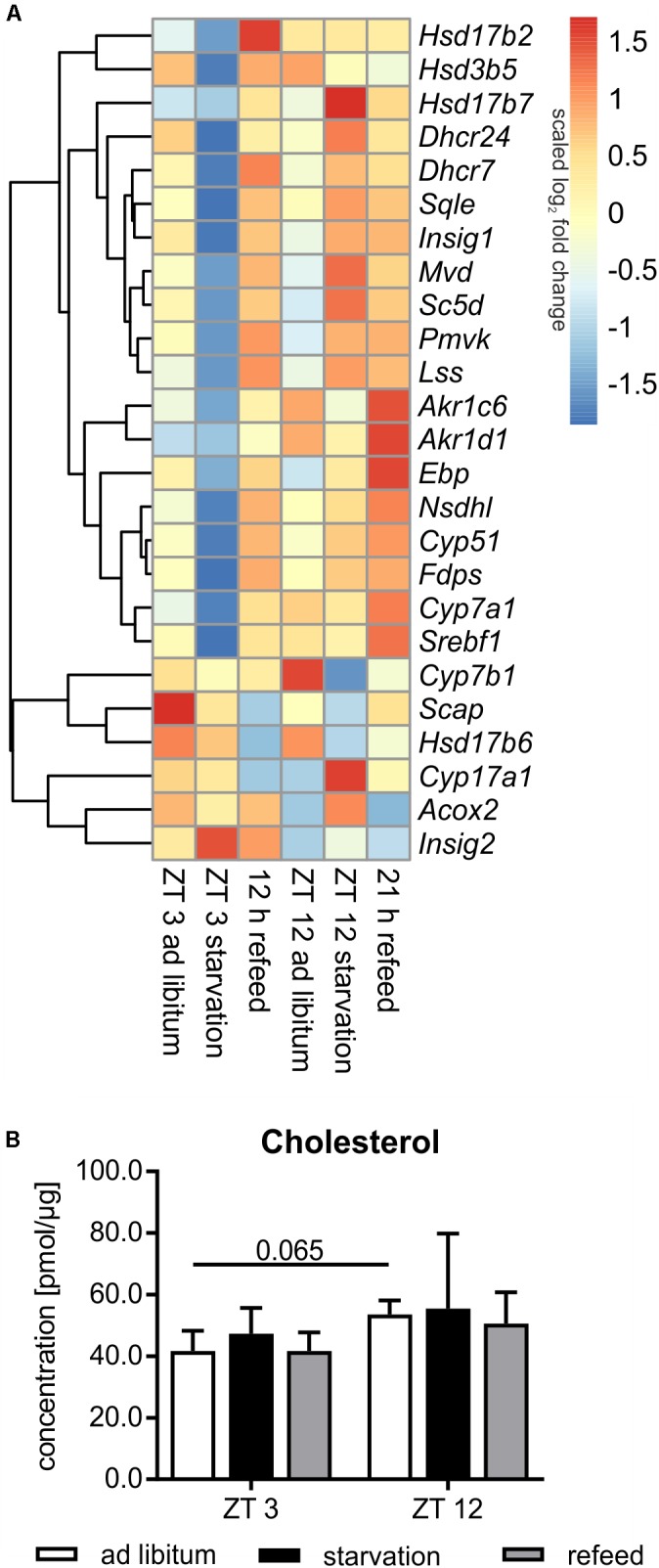
Regulation of steroid metabolism. **(A)** Heatmap of differentially expressed genes related to steroid metabolism. Red indicates an overexpression, and blue indicates an underexpression. **(B)** Concentration of cholesterol in primary hepatocytes of mice fed *ad libitum* (white bars), starved 24 h (black bars) or starved 24 h and refed 12 h (until ZT 3) and 21 h (until ZT 12) (gray bars) (*n* = 3). Data are plotted as mean ± standard deviation.

Steroid metabolism, especially cholesterol synthesis, is another important function of the liver, and it is partially regulated diurnally (**Figure 8A**). Comparing ZT 3 and ZT 12 *ad libitum Pmvk* (phosphomevalonate kinase), *Mvd* (mevalonate decarboxylase), *Ebp* (phenylalkylamine Ca^2+^ antagonist binding protein), *Sc5d* (sterol-C5-desaturase), and *Cyp17a1* (cytochrome P450, family 17, subfamily a, polypeptide 1) exhibited a higher expression in the morning than in the evening. The regulators of SREBP1, *Scap* (SREBP cleavage-activating protein) and *Insig1*/*2* (insulin induced gene), were also expressed higher at ZT 3 than at ZT 12. In accordance with enhanced synthesis over the day, the amount of hepatic cholesterol is 1.3-fold higher in the evening than in the morning (**Figure 8B**). Concerning steroidogenesis and cholesterol conversion, the hydroxysteroid dehydrogenases *Hsd3b5* and *Hsd17b2* showed a higher expression in the evening. The expression of many enzymes [*Cyp7a1*, *Cyp7b1*, *Akr1d1*, *Akr1c6* (aldo-keto reductase family 1, member D1/C6)] involved in steroid degradation by formation of bile acids were also higher expressed in the evening than in the morning.

##### Starvation

Already in the early fifties it was shown that the synthesis rate of cholesterol is markedly decreased by starvation. This metabolic profile was true in mice starved from ZT 3, in which most genes encoding cholesterol synthesizing enzymes [*Pmvk*, *Mvd*, *Fdps* (farnesyl diphosphate synthetase), *Sqle* (squalene epoxidase), *Lss* (lanosterol synthase), *Dhcr24* (24-dehydrocholesterol reductase), *Cyp51*, *Nsdhl* (NAD(P) dependent steroid dehydrogenase-like), *Hsd17b7*, *Ebp*, *Sc5d*, and *Dhcr7*] exhibited reduced expression compared to *ad libitum* mice (**Figure 8A**). By contrast, the shift of starvation time to the evening produced a reverse regulation and a primarily elevated expression of these genes. Notably, 24 h starvation did not significantly alter the cholesterol content (**Figure 8B**). The regulatory transcription factor of cholesterol synthesis, *Srebf1a*, was not significantly altered following starvation (**Figure 6**). Due to starvation initiated at ZT 3, *Insig1* and *Insig2* exhibited a reciprocal expression, reflecting published data (Ye and DeBose-Boyd, 2011), while the evening starvation period resulted in an equal increase in *Insig1* and *Insig2* expression. *Scap* expression was slightly lower after starvation in both periods (**Figure 8A**).

Cytochrome P450 enzymes (CYPs) and hydroxysteroid dehydrogenases (HSDs) synthesize steroid hormones with cholesterol as starting compound. The expression of *Cyp17a1* (steroid 17α-monooxygenase), which is a key enzyme of steroidogenesis, was highly induced after starvation started at ZT 12 and not altered in ZT 3 mice. By contrast, Hsd expression (*Hsd3b5*, *Hsd17b2*, and *Hsd17b6*) was more or less down-regulated after both starvation periods (**Figure 8A**).

The breakdown of cholesterol is performed by the synthesis of bile acids in the liver. *Cyp7a1*, which is the rate-limiting enzyme, was dramatically down-regulated following starvation initiated at ZT 3 and unchanged in ZT 12 mice compared to *ad libitum*. *Cyp7b1* was almost unaffected after starvation started at ZT 3 and highly decreased in ZT 12 mice. *Akr1c6* is another enzyme of bile acid synthesis and was down-regulated after both starvation periods. *Akr1d1* was marginally altered. *Acox2* was unchanged after starvation initiated at ZT 3, but it was increased in ZT 12 mice (**Figure 8A**).

Cholesterol metabolism exhibits a similar regulation in response to fasting like the lipid metabolism: cholesterol synthesis was regulated in the known manner in ZT 3 mice, but its synthesis exhibited unknown regulation in ZT 12 mice.

##### Refeeding

The genes of cholesterol synthesis (*Pmvk*, *Mvd*, *Sqle*, *Lss*, *Dhcr24*, *Hsd17b7*, and *Scd5*) remained elevated after the 21 h refeeding period compared to ZT 12 *ad libitum* (**Figure 8A**). *Fdps*, *Cyp51*, *Nsdhl*, and *Ebp* were even higher expressed after 21 h refeeding compared to the corresponding starvation period at ZT 12. The expression of almost all above-mentioned genes was elevated after the 12 h refeed compared to ZT 3 *ad libitum* and was very similar to that under ZT 12 starvation. Only *Ebp* and *Scd5* exhibited restored expression after 12 h refeeding (**Figure 8A**). Expression of the regulator *Srebf1a* was highly induced after the 12 h refeeding compared to ZT 3 *ad libitum*, and it was restored after refeeding for 21 h (**Figure 6**). *Scap* and *Insig2* exhibited a similar expression after 21 h refeed and at ZT 12 *ad libitum*. After 12 h refeed the expression of *Insig1* was slightly increased and *Insig2* was elevated much more, whereas *Scap* was decreased (**Figure 8A**).

*Cyp17a1* expression remained increased after the 21 h refeeding and decreased after 12 h compared to the corresponding *ad libitum* samples. *Hsd17b6* expression was lower after 12 and 21 h refeed than in the *ad libitum* samples. *Hsd3b5* remained down-regulated after 21 h refeeding. *Hsd17b2* expression increased after 12 h refeed (**Figure 8A**).

The key enzyme of bile acid synthesis *Cyp7a1* was up-regulated after both refeeding times. *Akr1d1* and *Akr1c6* were also up-regulated, especially after the 21 h refeed. *Acox2* expression was restored after refeeding (12 and 21 h), but *Cyp7b1* remained down-regulated after 21 h of refeeding (**Figure 8A**).

### Lipidome Analysis of Hepatocytes

**FIGURE 9:**
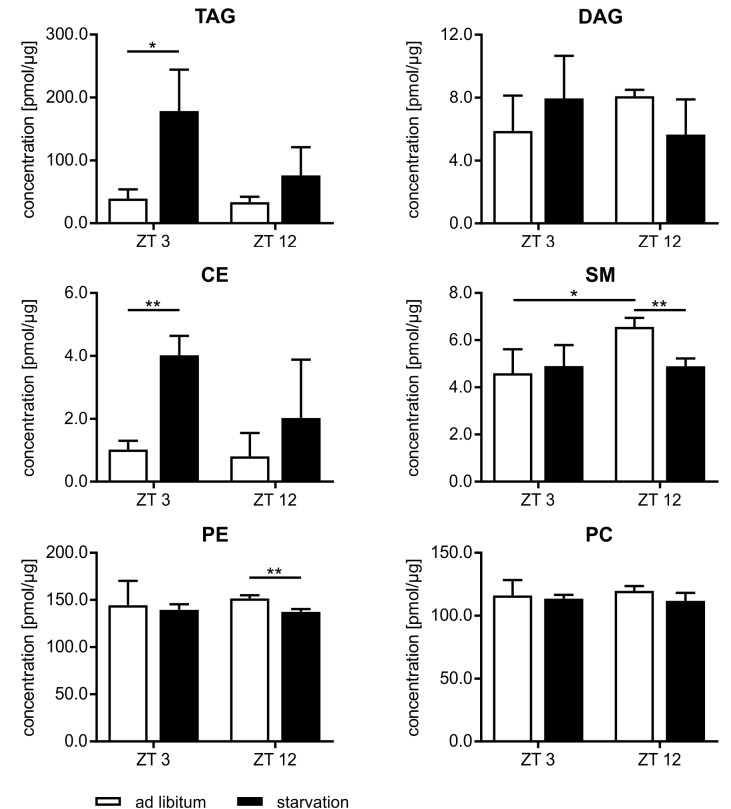
Lipidome profile of primary hepatocytes. Mice were fed *ad libitum* (white bars) or starved 24 h (black bars). Concentration of tri- and diacylglycerides (TAG/DAG), cholesteryl esters (CE), sphingomyelins (SM), phosphatidylethanolamines (PE), and phosphatidylcholines (PC) (*n* = 3). Data are plotted as mean ± standard deviation, ^∗^*p* < 0.05 and ^∗∗^*p* < 0.01.

Beside the evaluation of the gene expression, we analyzed the lipidome profile of the hepatocytes via mass spectrometry (**Figure 9**). The amount of lipids in mice fed *ad libitum* exhibited a basal level and no diurnal changes, with the exception of sphingomyelin (SM), where the content was 1.4-fold higher in ZT 12 than in ZT 3 mice. However, hepatic lipids used for storage, such as TAGs and cholesteryl esters (CEs), were strongly elevated in starving mice, and this regulation is highly time-dependent. Starvation started at ZT 3 produced a greater than fourfold increase in TAGs and CEs compared to *ad libitum* mice. By contrast, 24 h starvation initiated at ZT 12 produced only an approximately twofold increase. Polyunsaturated TAG species primarily reflected these differences in TAG levels (**Supplementary Figure 2**). However, TAGs with a high saturation level (50:1 and 52:2) were equally increased after both starvation periods because these TAGs are less reactive and remain longer in the cells. Diacylglycerides (DAGs) are transient precursor of TAG, and these molecules are produced at a much lower level. Both starvation periods slightly altered DAG levels. The membrane components SM and phosphatidylethanolamines (PE) were significantly reduced when starvation was initiated at ZT 12. The PC content did not change (**Figure 9**). Hepatic TAG exhibited still a 2.5-fold increase after the 12 h refeeding compared to *ad libitum* mice. However, the 21 h refeeding normalized the TAG concentration. Refeeding did not significantly alter the other lipid classes (**Supplementary Figure 3**).

## Discussion

**FIGURE 10:**
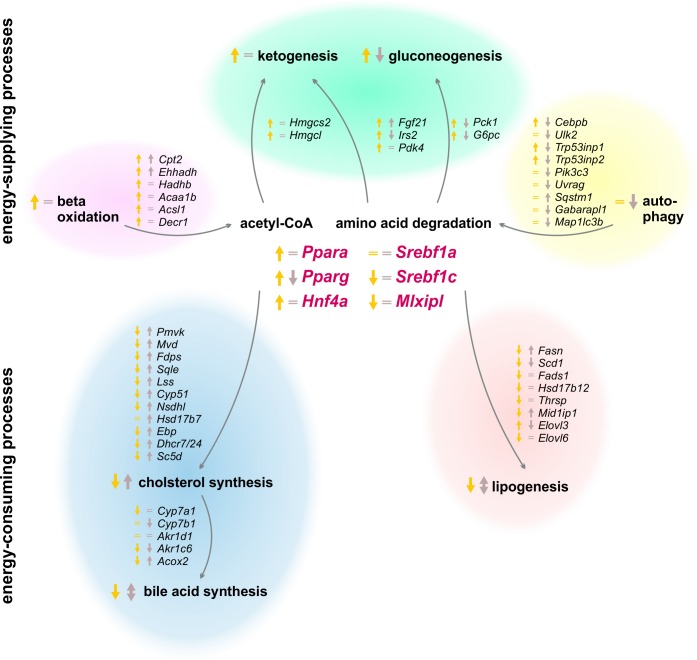
Summary of expression changes after 24 h starvation. Arrows indicate higher (↑) or lower (↓) expression after starvation initiated at ZT 3 (yellow) and ZT 12 (brown) compared to the corresponding *ad libitum* samples. Equality signs (=) indicate no expression changes after starvation.

The results of our study revealed a strong circadian-driven response to fasting in the liver (**Figure 10**). Twenty-four hour starvation initiated and terminated in the morning (ZT 3 to ZT 3) induced the expression of genes involved in metabolic pathways that produce energy-rich substrates for the organism. The gene expression of energy-consuming and temporary expendable processes diminished. However, starvation started in the evening (ZT 12 to ZT 12) produced a totally different hepatic expression signature, with partially opposing regulations, e.g., genes involved in gluconeogenesis decreased, while genes of fatty acid and cholesterol synthesis were induced. These novel findings were unraveled by the analysis of transcriptome data using SOMs. SOMs perfectly visualized the opposing expression profiles of the above-mentioned processes by comparing ZT 3 and ZT 12 starvation (clusters B and J, **Figure 2**). These differences in the expression of metabolic enzymes and their regulators are discussed below in detail.

### Metabolic Adaptions Upon Starvation

So far, it was assumed that starvation adapts liver metabolism in two ways: (i) by activating processes producing energy-rich metabolites and (ii) by suppressing energy-consuming pathways. However, our study revealed new diurnal-dependent aspects of these mechanisms. Acetyl-CoA and glucose or equivalents are essential energy-rich substrates produced by the liver upon starvation. Our study confirmed the strong induction of the expression of genes responsible for beta-oxidation, especially when starvation was initiated at ZT 3. However, this induction was much lower at ZT 12. Expression levels of essential enzymes of gluconeogenesis (*Pck1* and *G6pc*) and ketone body synthesis (*Hmgcs2* and *Hmgcl*) were distinctly decreased after food deprivation started in the evening and increased in the morning in the known way (Potthoff et al., 2009). Because both processes use similar starting compounds, IRS2 and PDK4 balance the rate of gluconeogenesis and ketone body synthesis in the liver upon fasting, respectively. Our microarray demonstrated increased *Irs2* and *Pdk4* expression when food deprivation began at ZT 3, as shown previously (Wu et al., 2000; Ide et al., 2004), whereas evening starvation did not affect *Pdk4* expression and decreased *Irs2*. Another process of delivering energy is autophagy, whereby, especially during starvation, expendable or dysfunctional cellular components are degraded and recycled (Yin et al., 2008). As a consequence of starvation started at ZT 12, however, the autophagic genes exhibited a lower expression level compared to ZT 12 *ad libitum* conditions. This result was consistent with the strongly decreased expression of a potent activator of autophagy, *Cebpb*, after starvation in the evening. In contrast, upon starvation in the morning, *Cebpb* exhibited induced expression, although most autophagic genes were not relevantly altered. It was published that the activation of autophagy is based on a changed phosphorylation pattern (Shang et al., 2011), but our results indicate a transcriptional regulation as well, which needs to be further investigated. The transcriptional data suggest a so far unknown down-regulation of energy-supplying processes after starvation started in the evening, while morning starvation led to the known activation of those pathways.

For energy-consuming and temporary expendable metabolic processes, we discovered similar diurnal regulation differences by starvation. The synthesis of fatty acids, manly carried out by fatty acid synthase, seems to be increased based on the elevated expression of *Fasn* after food deprivation in the evening. This observation was contrary to the decreased *Fasn* expression detected after starvation started in the morning and the published knowledge of diminished lipogenesis upon fasting (Horton et al., 1998). The hypothesis of the opposing lipogenesis regulation after different starvation periods was strengthened by the expression of *Gck*, forming the carbon source (pyruvate) for lipogenesis, which was also induced after starvation started in the evening and decreased in the known way after morning starvation (Iynedjian et al., 1987). Cholesterol synthesis is another process known to be diminished while starving. However, enzymes of cholesterol synthesis exhibited elevated gene expression following starvation initiated at ZT 12. Food deprivation initiated at ZT 3 reduced the expression levels of most down-stream genes encoding cholesterol synthesizing enzymes, which indicates diminished cholesterol synthesis, as previously shown (Tomkins and Chaikoff, 1952), even if expression of the rate-limiting enzyme of cholesterol synthesis, *Hmgcr* (3-hydroxy-3-methyl-glutaryl-coenzyme A reductase), was not found in our microarray. Bile acids are secreted into the intestine to increase the solubility of hydrophobic molecules and allow their absorption (Hofmann and Borgström, 1964) and have a poorly understood function as potent signaling compounds (Chiang, 2017). Our study revealed a higher expression of genes involved in bile acid synthesis in the evening than in the morning in *ad libitum* fed mice. This result was consistent with the known diurnal expression of the rate-limiting enzyme CYP7A1, which is highest when the greatest amount of food is consumed (Gooley, 2016). Mice are nocturnal and consume approximately three times more food during scotophase than during photophase (Kurokawa et al., 2000). Bile acid production is a redundant process during starvation, and our analysis revealed a lower expression of *Cyp7a1* after food deprivation at ZT 3. However, starvation in the evening did not alter *Cyp7a1* expression. Several groups reported a similar pattern of *Cyp7a1* expression after starvation (Noshiro et al., 1990; Li et al., 2012), but other studies demonstrated an induction (De Fabiani et al., 2003; Shin et al., 2003). The timing and length of the starvation period used by different groups may explain these diverse results and illustrate the importance of our study. Steroid hormone synthesis is generally localized in the gonads and adrenal glands, but the adult liver also performs steroidogenesis under specific conditions (Grasfeder et al., 2009; Rennert et al., 2017). *Cyp17a1* expression, which is a central steroidogenic enzyme, was induced following evening starvation. This result confirms previous work and strengthens the idea of steroids as mediators of starvation responses (Bauer et al., 2004; Grasfeder et al., 2009). Our data show a novel regulation of energy-consuming processes after starvation started in the evening, contrary to the established down-regulation frequently published as the hepatic starvation response after fasting initiated in the morning.

The accumulation of lipids in the liver (steatosis) often accompanies starvation (Kok et al., 2003; van Ginneken et al., 2007). Adipose tissues secrete fatty acids, which are taken up by hepatocytes and esterified to the storage lipids TAG and CE or secreted as VLDL. Lipidomics analysis revealed significantly elevated TAG and CE contents due to starvation, but this increase was twice as great after food deprivation initiated in the morning than when it was initiated in the evening. This may be based on the diurnal regulation of adipose triglyceride lipase (ATGL) and hormone-sensitive lipase (HSL), which are the lipolysis pacemaker enzymes in adipose tissue and direct targets of CLOCK/ARNTL (Shostak et al., 2013). The lower level of TAG and CE after starvation started in the evening appears inconsistent with the increased expression of *Fasn*. Two possible explanations are (i) since *Fasn* was only detected at the mRNA level, the synthesis of enough enzyme protein and the accumulation of a measurable increase of TAG may be delayed for several hours and/or (ii) the synthesized fatty acids were not stored in the liver but secreted, and the analysis could not capture them. To unravel this uncertainty, additional studies have to be performed.

### Regulation of the Hepatic Starvation Response

Additionally, for the transcription factors regulating the observed starvation-induced transcriptional alterations, our study exhibited novel diurnal expression profiles. A main activator of the energy-supplying processes is the PPAR family. Elevated expression of *Ppara* and *Pparg* in the liver upon fasting induces beta-oxidation, stimulating lipid uptake and fatty acid storage (Kersten et al., 1999; Gavrilova et al., 2003; Tanaka et al., 2005). However, we detected this up-regulated expression only when starvation was initiated at ZT 3 and not at ZT 12, which explains the weak induction of beta-oxidation, the decreased gluconeogenesis and ketogenesis and the lower amount of TAG accumulation in the liver after evening starvation. Furthermore, HNF4A contributes to this regulation due to additional activation of *Ppara* and diminishment of the repressor *Hes6* (Martinez-Jimenez et al., 2010). Our data nicely reflected this mechanism after starvation started in the morning. However, after evening starvation, *Hnf4a* was unchanged and the *Hes6* level increased.

The SREBP1 family and ChREBP (*Mlxipl*) are transcription factors more responsible for the post-prandial state and are supposed to be down-regulated during starvation to not activate energy-consuming pathways. For *Mlxipl*, we detected a transcriptional response with a strong down-regulation after starvation in the morning, but no changes were observed when starvation was initiated in the evening. This was contrary to the published regulation carried out only at the post-translational level via phosphorylation (Iizuka and Horikawa, 2008) and requires a more focused study. *Srebf1c* expression was reduced only after food deprivation initiation in the morning, and its expression was unchanged following starvation in the evening, which may explain the induced expression of the target *Fasn* when starvation was started at ZT 12. SREBP-1a is known to regulate cholesterol synthesis, but even if its expression was not relevantly altered, neither after starvation initiated in the morning nor in the evening, the genes encoding cholesterol synthesizing enzymes responded with a down- or up-regulation, respectively. Additionally, the influence of the SREBP1 inhibitors, INSIG1/2, seemed to depend on diurnality, since *Insig1*/*2* expression was elevated after both starvation periods, but the regulatory output differed. Therefore, the known regulatory mechanisms of *Srebf1* expression did not seem applicable when starvation was initiated in the evening, and further investigations are needed to delineate these mechanisms. All in all, the diurnally regulated expression of the transcription factors *Ppara*, *Pparg*, *Mlxipl*, *Srebf1a*, and *Srebf1c* following starvation likely underlie many of the observed metabolic alterations.

Concerning the regulation of the fed and starved state, two hormones are omnipresent: insulin and glucagon. Since the hormones were not determined in our study, we can only speculate about their levels and influence. Shi et al. (2013) demonstrated diurnal differences in mice with enhanced insulin activity during the night and a metabolism that was characterized by insulin resistance during the day. However, 24 h starvation started in the morning changed the diurnal insulin regulation, and serum insulin levels dropped by approximately two-thirds, resulting in the known starvation responses (Ahrén and Havel, 1999), but no data were available for evening starvation. Glucagon, on the other hand, regulates metabolism in the fasted state. It was shown that glucagon induces the expression of the hormone *Fgf21* (Berglund et al., 2010), which in turn activates gluconeogenesis and ketogenesis. The expression of *Fgf21* was much more induced when starvation was started in the morning than in the evening, which explains the diurnal differences in gluconeogenic and ketogenic gene expression and suggests that the glucagon response also depends on the timing of starvation.

The overall regulators of circadian rhythm are the core clock genes *Arntl*, *Clock* and *Per*, which were elevated after both starvation periods but maintained their typical diurnal expression pattern. This result was consistent with a previous study that demonstrated induced *Arntl* expression due to raised glucagon levels (Sun et al., 2015). Since the transcriptional analysis was performed in hepatocytes, a screen of other organs would help to fully understand the regulatory differences following starvation started in the morning and in the evening.

### Metabolic Adaptions and Regulations Upon Refeed

**FIGURE 11:**
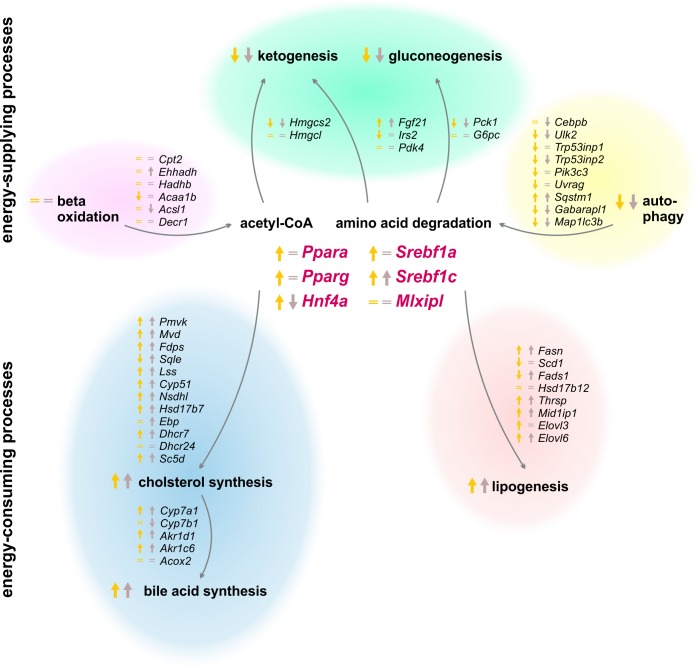
Summary of expression changes following 12 and 21 h refeeding after 24 h starvation. Arrows indicate higher (↑) or lower (↓) expression after refeeding for 12 h until ZT 3 (yellow) and 21 h until ZT 12 (brown) compared to the corresponding *ad libitum* samples. Equality signs (=) indicate a restored *ad libitum* expression levels.

Peripheral tissues first refill their glucose stores when an organism switches from a starved to a refed state, and the liver subsequently synthesizes and stores glycogen, fatty acids, and cholesterol (Berg et al., 2002). Our experimental setting investigated the effects of two refeeding durations (12 and 21 h) after the same starvation period (**Figure 11**). The already mentioned clusters B and J in the SOMs, where most genes of intermediary and steroid metabolism were localized, exhibited similar expression profiles after both refeeding periods, which were totally different from the *ad libitum* groups. The expression levels in other regions of the SOM after 21 h refeeding partially resembled the *ad libitum* group, which indicated a return to the untreated state.

Since food intake delivers all essential metabolites, the liver (i) activates the energy-consuming metabolism and (ii) the production of energy-rich substrates returns to normal. The liver no longer synthesizes ketone bodies and glucose after refeeding, and the expression of the relevant genes reached *ad libitum* or decreased levels. The genes of beta-oxidation were expressed at *ad libitum* levels as well. Early findings demonstrated that refeeding suppressed autophagy (Mortimore et al., 1983), which was confirmed since most of the autophagic genes exhibited a much lower expression after 12 h refeeding compared to starvation, and these genes returned to an approximately *ad libitum* level after 21 h. In contrast, the synthesis of fatty acids and steroids is induced by refeeding. Our study demonstrated that the expression of *Fasn* was highly increased to a maximum after the 21 h refeed. The genes encoding cholesterol synthesizing enzymes were elevated after 12 and 21 h of refeeding as well. This led to an induced synthesis of fatty acids and cholesterol to refill the emptied stores. Bile acid synthesis is associated with the food consumption, and it increased due to elevated expression levels of *Cyp7a1*, as reported previously (Li et al., 2012). The lipidome profile revealed that TAG content remained increased after 12 h refeeding but reached *ad libitum* levels after 21 h. Previous studies demonstrated a normalization of liver lipid content after 48 h of refeeding (Kok et al., 2003).

Since SREBF1 stimulates lipogenesis and cholesterol synthesis, the expression of the transcription factors *Srebf1a* and *Srebf1c* was highly induced after 12 h refeeding, which was consistent with previous results (Horton et al., 1998; Ide et al., 2004) and resulted in the observed induction of fatty acid and cholesterol synthesis. Resulting from differences in the timing of applied starvation periods, our results revealed another profile than what has been suggested thus far for the expression of the inhibitors *Insig1* and *Insig2*. We demonstrated that *Insig1* levels were almost unchanged and *Insig2* remained highly elevated after 12 h refeeding compared to the corresponding ZT 12 starvation group, contrary to the published decrease of *Insig1* and *Insig2a* expression after refeeding (Attie, 2004; Lee et al., 2017). This discrepancy illustrates the importance of further knowledge of the circadian-driven response to fasting in the liver. The transcription factors, *Ppara* and *Pparg*, remained elevated after the 12 h refeeding but reached *ad libitum* levels after 21 h. Geisler et al. (2016) demonstrated a similar decline in *Ppara* levels with advanced refeeding times.

### Linkage of Starvation and Refeed

SOMs that the overall hepatic expression after 12 h refeeding highly resembled starvation initiated at ZT 12, which was confirmed according to the details of different metabolic pathways (e.g., lipogenesis, cholesterol synthesis, autophagy). This may be a consequence of the nocturnal eating behavior of mice, because the expression of many genes may be decreased as a direct consequence of starvation when mice were sacrificed after 24 h starvation in the morning directly after the scotophase when they typically consume their major meal. By contrast, when mice are sacrificed in the evening, the previous period was the photophase during which they consume less food, and the diurnal regulation of mice anticipating food may predominate. Furthermore, mice in which starvation was started in the evening consumed only small amounts of food in the prior period because it was daytime, and the fasting period was actually longer and the stores may be more depleted. These differences may also explain the differences in TAG content. We can only speculate on these points because refeeding in our experiments was only initiated in the evening and not in the morning. We also cannot distinguish between the effects of the longer refeeding period and circadian regulation because samples were taken during another circadian time.

## Conclusion

Our experiments convincingly demonstrate that the response to starvation periods differed depending on the timing of starvation initiation and report valuable new information about expression levels based on the initiation and termination of starvation in the evening. Performing analogous experiments in humans may provide useful information of the metabolic state after differently timed starvation periods, which may lead to better understanding stressful starvation conditions and develop medical treatment strategies for affected patients. In chronic disturbances of meal timing and the circadian rhythm, e.g., upon shift work, various metabolic disorders are known (Canuto et al., 2013; James et al., 2017). The diverse timings and lengths of starvation periods used in different research groups may explain the variance in published results and support the necessity for clearly designed and recorded experimental procedures. Accordingly, the circadian influence must be considered in all *in vivo* experiments including starvation.

## Author Contributions

CR performed the experiments and analyzed the data. SV performed the bioinformatical analysis. EM-B and CT collected the samples. SS and AS performed the lipidomics analysis. RG designed the study. CR, SV, EM-B, RG, and MM-S wrote or edited the manuscript.

## Conflict of Interest Statement

The authors declare that the research was conducted in the absence of any commercial or financial relationships that could be construed as a potential conflict of interest.

## Footnotes

1 https://seek.lisym.org/data_files/98?code=YGHGwNPpYhqpiEBnnv4qb%2BchjgjUoYGhH6bc3OQp

2 https://david.ncifcrf.gov/

3 http://string-db.org
