# Supplementary material for: The Diurnal Timing of Starvation Differently Impacts Murine Hepatic Gene Expression and Lipid Metabolism – A Systems Biology Analysis Using Self-Organizing Maps
Source: Front Physiol. 2018 Sep 10;9:1180. doi: 10.3389/fphys.2018.01180 (PMC6146234; doi:10.3389/fphys.2018.01180)
Supplement: Supplementary file 3 [file Data_Sheet_1.pdf]

Supplementary Material

The Diurnal Timing of Starvation Differently Impacts Murine Hepatic Gene Expression and Lipid Metabolism – A Systems Biology Analysis Using Self-Organizing Maps

Christiane Rennert[1], Sebastian Vlaic[2], Eugenia Marbach-Breitrück[1,3], Carlo Thiel[1], Susanne Sales[4], Andrej Shevchenko[4], Rolf Gebhardt[1], Madlen Matz-Soja[1]*

[1] Rudolf-Schönheimer-Institute of Biochemistry, Faculty of Medicine, Leipzig University, Leipzig, Germany

[2] Leibniz Institute for Natural Product Research and Infection Biology, Hans-Knöll-Institute, Jena, Germany

[3] Institute of Biochemistry, Charité – Universitätsmedizin Berlin, corporate member of Freie Universität Berlin, Humboldt-Universität zu Berlin, and Berlin Institute of Health, Berlin, Germany

[4] Max Planck Institute of Molecular Cell Biology and Genetics, Dresden, Germany

*** Correspondence:** Madlen Matz-Soja: madlen.matz@medizin.uni-leipzig.de

1 Supplementary Figures and Tables

1.1 Supplementary Figures

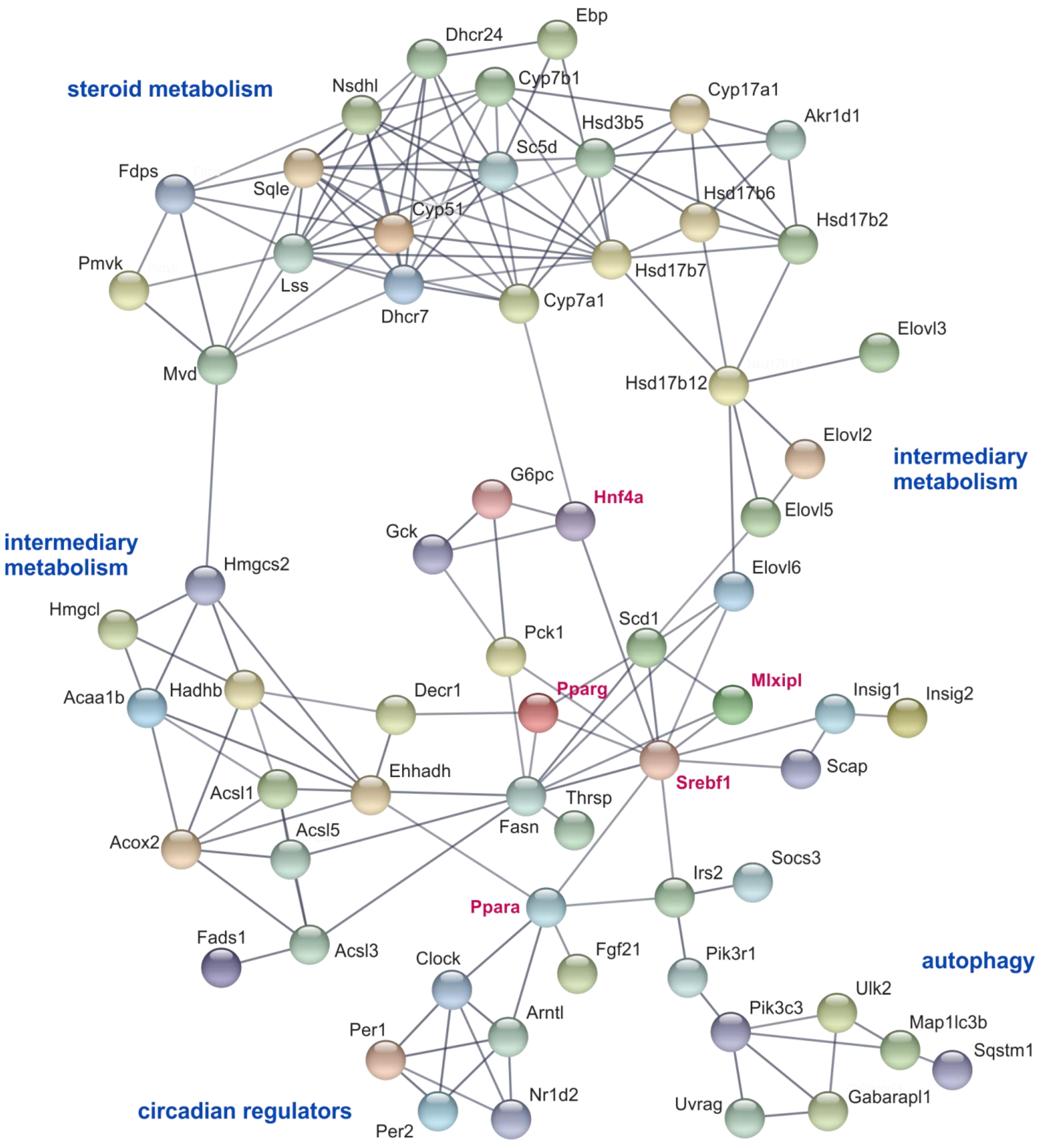

Supplementary Figure 1. The protein interaction network obtained from the STRINGv10.5 database displays the connections of the studied genes/proteins related to hepatic starvation response. The related pathways are marked in blue, transcription factors are marked in pink. The networks were constructed in the "confidence" mode with a high confidence score (0.7).

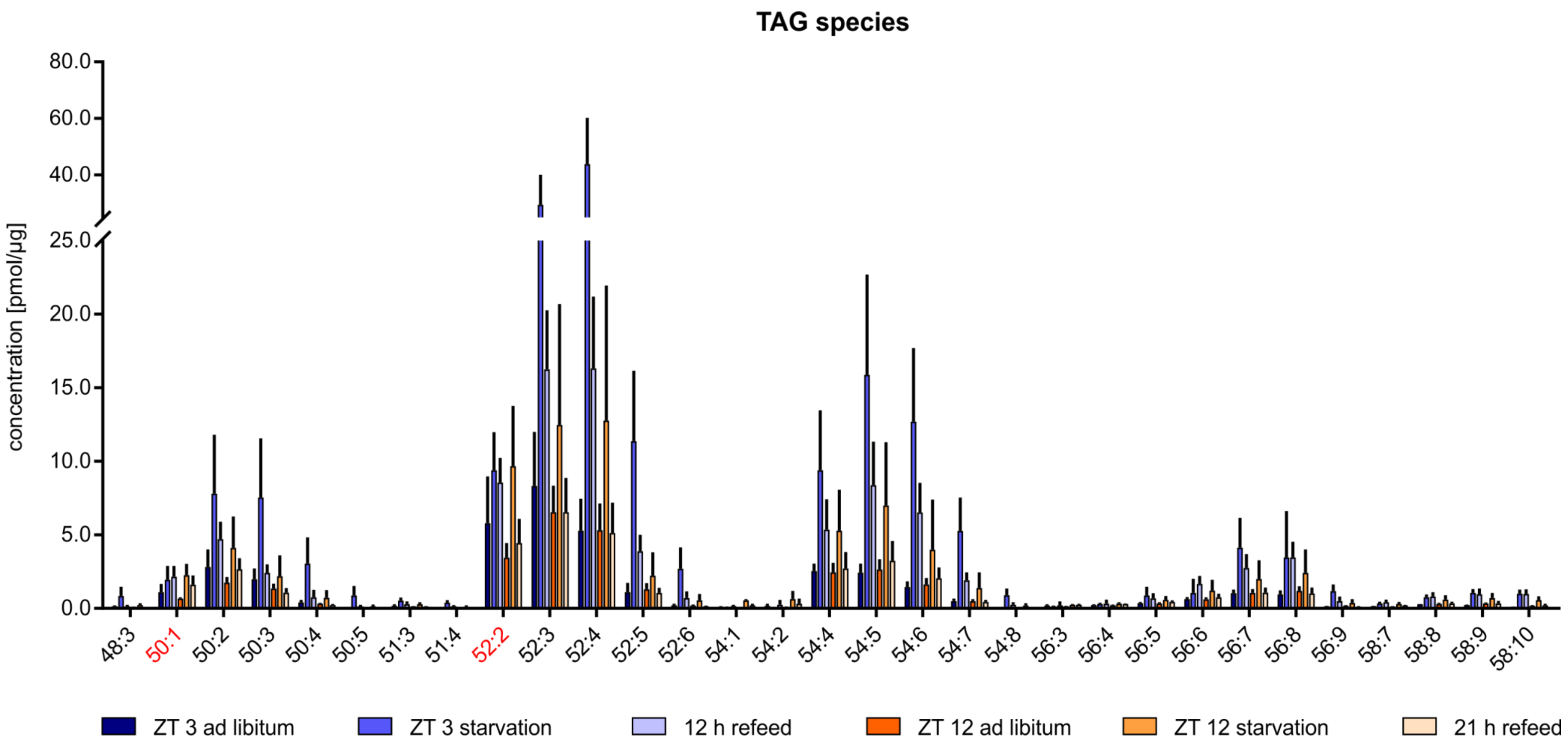

Supplementary Figure 2. TAG species in hepatocytes. Lipidomics analysis of primary hepatocytes of mice fed ad libitum, starved 24 h or starved 24 h and refed 12 and 21 h prior to sacrifice at ZT 3 or ZT 12. All detected TAG species are named with the total number of carbon atoms and double bonds in the fatty acids. Data are plotted as mean ± standard deviation.

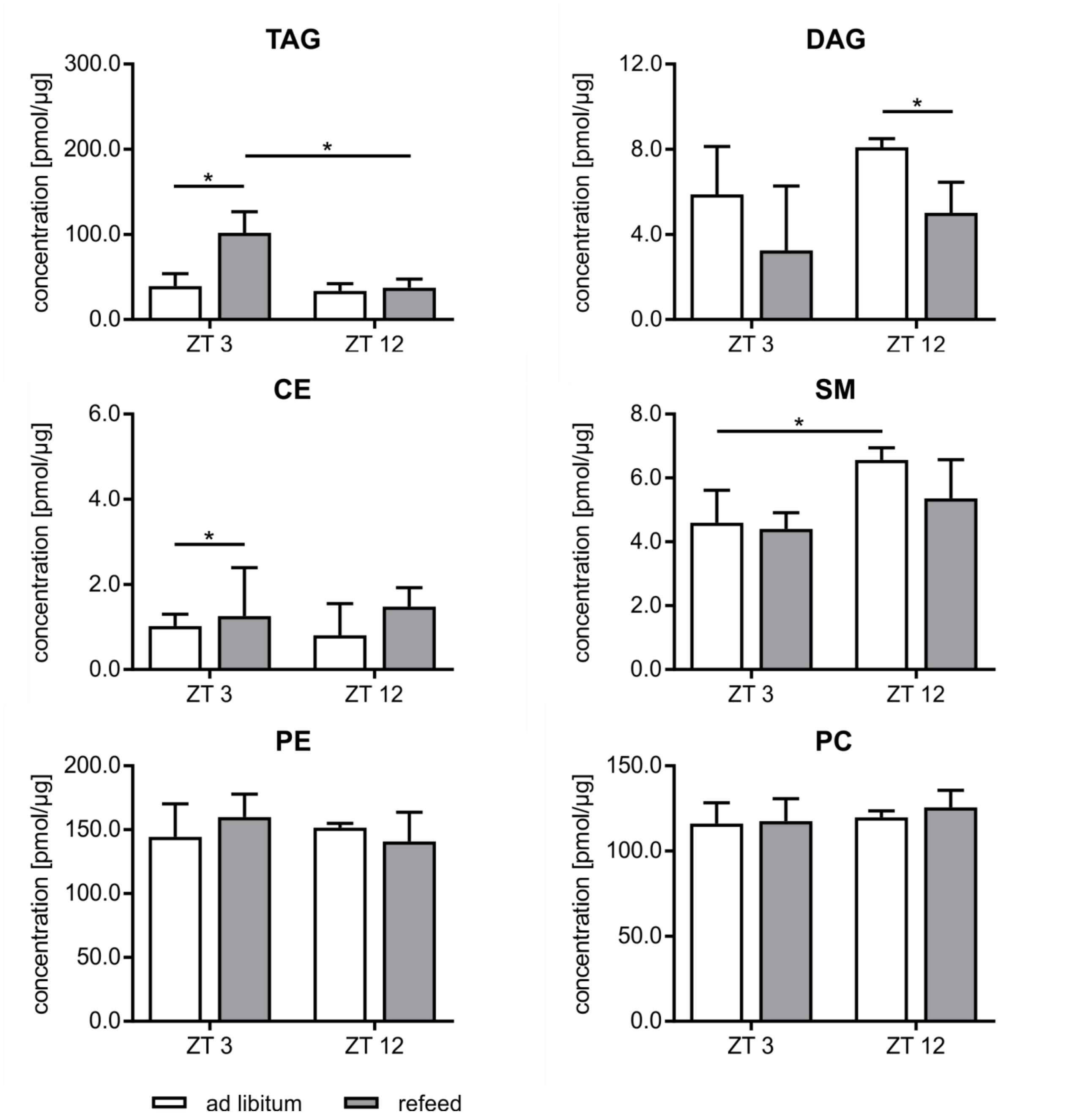

Supplementary Figure 3. Lipidome profile of primary hepatocytes. Mice were fed ad libitum (white bars) or starved 24 h and refed 12 h (until ZT 3) and 21 h (until ZT 12) (grey bars). Concentration of tri- and diacylglycerides (TAG/DAG), cholesteryl esters (CE), sphingomyelins (SM), phosphatidylethanolamines (PE) and phosphatidylcholines (PC) ($n = 3$). Data are plotted as mean ± standard deviation, $p < 0.05$ (*).

1.2 Supplementary Tables

Supplementary Table 1. Primer sequences for qPCR analyses.

gene	forward primer	reverse primer
18S	gcaattattccccatgaacg	gggacttaatcaacgcaagc
Fgf21	agatggagctctctatggatcg	gggcttcagactggtacacat
Ppara	cgtacggcaatggctttatc	tcatctggatggttgctctg
Pparg	atggaagaccactcgcattc	gctttatccccacagactcg
Srebf1a	cagacactggccgagatg	aaacaggcccgggaagtc
Srebf1c	gagccatggattgcacatttg	aggccagagaagcagaagag

Supplementary Table 2. (**A**) Gene-enrichment analysis (GEA) of all genes with an absolute expression change ≥ 1.5-fold. (**B**) Annotation of the SOMs by k-means clustering and following gene-enrichment analysis. List of all GO terms with Benjamini-Hochberg corrected p value < 0.05. BP = Biological Process, MF = Molecular Function, CC = Cellular Component

see separate Excel file ‘Supplementary Table 2’

Supplementary Table 3. Regulated genes detected by Illumina microarrays. The list contains $\log_2$ expression values of all genes with an absolute expression change ≥ 1.5-fold [$\log_2(1.5)$] between at least two of the six groups. Hepatocytes were isolated from mice fed ad libitum, starved 24 h or starved 24 h and refed 12 and 21 h prior to sacrifice at ZT 3 or ZT 12.

see separate Excel file ‘Supplementary Table 3’
